# Inhibition of mTOR by Rapamycin Results in Auditory Hair Cell Damage and Decreased Spiral Ganglion Neuron Outgrowth and Neurite Formation *In Vitro*

**DOI:** 10.1155/2015/925890

**Published:** 2015-03-31

**Authors:** Katharina Leitmeyer, Andrea Glutz, Vesna Radojevic, Cristian Setz, Nathan Huerzeler, Helen Bumann, Daniel Bodmer, Yves Brand

**Affiliations:** ^1^Department of Biomedicine, University Hospital Basel, Hebelstrasse 20, 4031 Basel, Switzerland; ^2^Clinic for Otolaryngology, Head and Neck Surgery, University Hospital Basel, Petersgraben 4, 4031 Basel, Switzerland

## Abstract

Rapamycin is an antifungal agent with immunosuppressive properties. Rapamycin inhibits the mammalian target of rapamycin (mTOR) by blocking the mTOR complex 1 (mTORC1). mTOR is an atypical serine/threonine protein kinase, which controls cell growth, cell proliferation, and cell metabolism. However, less is known about the mTOR pathway in the inner ear. First, we evaluated whether or not the two mTOR complexes (mTORC1 and mTORC2, resp.) are present in the mammalian cochlea. Next, tissue explants of 5-day-old rats were treated with increasing concentrations of rapamycin to explore the effects of rapamycin on auditory hair cells and spiral ganglion neurons. Auditory hair cell survival, spiral ganglion neuron number, length of neurites, and neuronal survival were analyzed *in vitro*. Our data indicates that both mTOR complexes are expressed in the mammalian cochlea. We observed that inhibition of mTOR by rapamycin results in a dose dependent damage of auditory hair cells. Moreover, spiral ganglion neurite number and length of neurites were significantly decreased in all concentrations used compared to control in a dose dependent manner. Our data indicate that the mTOR may play a role in the survival of hair cells and modulates spiral ganglion neuronal outgrowth and neurite formation.

## 1. Introduction

Until recently, sensorineural hearing loss due to degeneration and death of auditory hair cells (HCs) and their associated spiral ganglion neurons (SGNs) has been regarded as an inevitable consequence of age, genetic conditions, or exposure to certain environmental stimuli. Up-to-date, there is no treatment option available to cure sensorineural hearing loss and only prostheses such as hearing aids and cochlear implants offer some help. However, some of the intracellular pathways that mediate damage to HCs were discovered by using ototoxic agents, such as aminoglycoside to induce HC death* in vitro* [1–3] during the past several years. Aminoglycoside exposure activated some proapoptotic signaling pathways like the c-Jun-N-terminal kinase pathway and small GTPases, such as Ras and Rho/Rac/Cdc42 [4–6]. After prolonged aminoglycoside exposure, caspases are activated and HCs undergo apoptotic cell death [7, 8]. However, several pathways enhancing HC survival have also been discovered, such as the phosphatidylinositol-3-kinase (PI3K)/Akt [9] and H-Ras/Raf/MEK/Erk [6] pathways.

The mammalian target of rapamycin (mTOR) is a 290 kDa intracellular serine/threonine kinase. The mTOR pathway is an important regulator of cell growth, cell proliferation, cell metabolism, cell division, cell survival, and responses to cellular stress. In mammalian cells, two independent mTOR complexes (mTORC1 and mTORC2, resp.) have been characterized each of which contains the unique adaptor protein raptor or rictor, respectively [10]. mTORC1 is rapamycin sensitive and phosphorylates ribosomal protein S6 kinase and the translation repressor 4EBP1. In contrast, mTORC2 is insensitive to rapamycin and phosphorylates Akt by phosphorylation on Ser473 [11]. PI3K is one of the key activators of the mTOR pathway by activation of the serine/threonine kinase Akt and promotes cell growth and insulin-mediated effects on metabolism [12]. The tuberous sclerosis complex (TSC1/TSC2) tumor suppressors are critical upstream inhibitors of the mTORC1 complex. Loss of TSC1 or TSC2 leads to hyperactivation of the mTOR pathway and results in inherited cancer syndromes like Cowden's disease, neurofibromatosis type I, tuberous sclerosis complex, and Peutz-Jeghers syndrome, collectively called as phakomatoses [12]. Moreover, research demonstrated that mTOR dysregulation plays an important role in the genesis of various cancers like renal cell carcinoma, neuroendocrine tumor, gastric cancer, and hepatocellular carcinoma [13, 14]. As a consequence, mTOR became an important target for tumor gene therapy.

Rapamycin (brand name, Sirolimus) is a macrolide produced by the bacterium* Streptomyces hygroscopicus *found in the 1970s in the soil of Easter Island [11]. It is an antifungal agent with immunosuppressive effect. Rapamycin is a pharmacological inhibitor of mTOR and is highly specific for mTORC1 complex. After building a complex with its intracellular cofactor cyclophilin FKBP12, this complex interacts with mTORC1 and inhibits its function [15]. Due to its immunosuppressive effect, rapamycin is used to prevent rejection in organ transplantation. Moreover, rapamycin analogs (so-called rapalogs) are being used as anticancer drugs and clinical trials are conducted for the treatment of sarcomas and hematologic and solid malignancies [16].

During the last decade, various* in vivo *and* in vitro *studies have been conducted to understand the effect of rapamycin. Harrison et al. [17] demonstrated that the systemic administration of rapamycin extends life span and Halloran et al. [18] observed an anxiolytic and antidepressive effect of rapamycin in mice. Interestingly, rapamycin attenuates light-induced injury in retinal photoreceptor cells indicating a neuroprotective effect of this drug [19]. However, antiproliferative effects were also observed. Paliouras et al. [20] showed a reduced neuronal stem cell proliferation after rapamycin infusion into the left ventricle in mice and systemic administration of rapamycin reduced the number of neural progenitor cells in adult mammalian brain by inhibition of proliferation and differentiation of neural stem cells [21].

Little is known about the role of mTOR in the inner ear. Fang and Xiao [22] demonstrated that rapamycin alleviated cisplatin-induced ototoxicity* in vivo* by decreasing oxidative stress and promoting autophagy. Given the diverse effect of rapamycin on cell survival and proliferation in different systems, the aim of this study is to expand our knowledge of the role of mTOR in the inner ear. First, we evaluated the expression of the two independent mTOR complexes (mTORC1 and mTORC2, resp.) in the cochlea by performing western blotting of the unique adaptor protein raptor or rictor, respectively. This was done in cochleae exposed to rapamycin compared to controls without exposure to rapamycin. Next, we determine the effect of mTOR inhibition by rapamycin on HC survival. Finally, we evaluated the effects of rapamycin on SG neurite outgrowth, neurite formation, and survival* in vitro.*

## 2. Materials and Methods

### 2.1. Animal Procedures

All animal procedures were performed in Basel, Switzerland, according to an animal research protocol approved by the Committee on the Ethics of Animal Experiments of Basel (Kantonales Veterinäramt Basel, permit number: 2263), in accordance with the European Communities Council Directive of 24 November 1986 (86/609/EEC). Animals were sacrificed prior to all tissue extractions. For all experiments 5-day-old Wistar rats were used (Harlan, Netherlands).

### 2.2. Western Blotting

Animals were decapitated and cochleae were carefully microdissected in ice-cold PBS. Brain extract was used as a positive control. Cochlea were initially incubated in culture medium (Dulbecco's modified Eagle's medium (DMEM)) supplemented with 10% fetal calf serum (FCS), 25 mM HEPES, and 30 U/mL penicillin (Invitrogen, Carlsbad, CA, USA) for 24 hours at 37°C with 5% CO_2_ for recovery. After that period the OCs were treated with increasing amounts of rapamycin dissolved in DMSO (Sigma-Aldrich, St. Louis, USA) at final concentrations of 10 *μ*M and 100 *μ*M in the cell culture medium for 24 hours at 37°C with 5% CO_2_. Control OCs were incubated for 24 hours in culture medium without rapamycin in DMSO only. Explants were homogenized in CelLytic buffer containing protease inhibitor cocktail (Sigma-Aldrich, St. Louis, USA) and centrifuged. Supernatants were aspirated and placed in a new tube. Protein concentration was determined using the BCA Protein Assay Reagent kit (Pierce, Rockford, USA) according to the manufacturer's instructions. Lysates were mixed with Laemmli sample buffer and heated at 95°C for 5 min. A 20 *μ*g sample of each lysate was resolved on a 10% and 7% SDS-PAGE gel and blotted onto a polyvinylidene fluoride membrane. The nonspecific sites of the transferred proteins were blocked with blocking solution (Roche, Switzerland) diluted (1 : 100) in PBS-T (1.54 mM KH_2_PO4; 155.17 mM NaCl; 2.71 mM Na_2_HPO4-7H_2_O, pH 7.2; 0.1% Tween-20) for 1 h at room temperature. The membrane was washed with PBS-T (3 × 10 min) and then incubated with primary antibodies in TBS. The following primary antibodies were used: rabbit polyclonal anti-Raptor and anti-Rictor (1 : 1000, Cell Signaling, Switzerland) and mouse monoclonal anti-*β*-actin (1 : 5000, Santa Cruz Biotech, California, USA). The membranes were incubated with the primary antibodies overnight at 4°C. Next, the blots were incubated with monoclonal anti-rabbit peroxidase-conjugated secondary antibody (1 : 80,000; Sigma-Aldrich, Switzerland) and anti-mouse peroxidase-conjugated secondary antibody (1 : 200,000; Sigma-Aldrich, Switzerland).

The blots were washed with PBS-T (3 × 10 min) and incubated with appropriate POD-conjugated secondary antibody for 1 h at room temperature (Roche, Switzerland). After washing, bands were visualized using BM chemiluminescence developmental solution (Roche, Switzerland). The immunopositive bands were visualized by autoradiography. Serial exposures were made to radiographic film (Hyperfilm ECL; Amersham Biosciences).

### 2.3. OC Tissue Culture

Five-day-old Wistar rat pups (Harlan, Netherlands) were decapitated. Cochlear microdissections were performed under a light microscope to isolate the OC and the spiral ganglion (SG) as described by Sobkowicz et al. [23]. OCs were initially incubated in culture medium (Dulbecco's modified Eagle's medium (DMEM)) supplemented with 10% fetal calf serum (FCS), 25 mM HEPES, and 30 U/mL penicillin (Invitrogen, Carlsbad, CA, USA) for 24 hours at 37°C with 5% CO_2_ for recovery. After that period the OCs were treated with increasing amounts of rapamycin dissolved in DMSO (Sigma-Aldrich, St. Louis, USA) at final concentrations of 10 *μ*M, 50 *μ*M, and 100 *μ*M in the cell culture medium for 48 hours at 37°C with 5% CO_2_. Control OCs were incubated for 48 hours in culture medium without rapamycin in DMSO only.

### 2.4. HC Count and Statistical Analysis

OCs were fixed in 4% paraformaldehyde and permeabilized with 5% Triton X-100 in phosphate-buffered saline (PBS). Then the OCs were incubated with an anti-myosin VIIa antibody solution containing an anti-myosin VIIa rabbit antibody (1 : 500, Abcam, UK) in 1% bovine serum albumin (BSA)/PBS blocking solution for 2 hours at room temperature. After washing with PBS three times, the OCs were incubated in PBS containing the secondary antibody, fluorescent-labeled Alexa TM 488 (1 : 250, Molecular Probes, LuBio Science, Switzerland), for 1 hour at room temperature. The OCs were fixed and photographed using a fluorescence microscope (Olympus BX63, Center Valley, PA, USA).

Evaluation of 60 OHCs associated with 20 IOCs in a given microscopic field was performed for quantitative analysis. The analysis included the apical, the middle, and the basal turn, with three random microscope fields counted in each explant per cochlear turn. These values were then averaged across the six replications of each experiment. Inner HCs and outer HCs were counted and used to assess HC survival. HC counting results were analyzed by analysis of variance (ANOVA), followed by the least significant difference (LSD) post hoc test (Stat View 5.0). Differences associated with *P* values of <0.05 were considered to be statistically significant. All data are presented as mean ± SD.

### 2.5. Preparation of Tissue Culture Plates for Rat SGN Experiments

24-well cell culture plates (Costar, Corning Inc., Acton, MA, USA) were uniformly coated with 300 *μ*L of 5 *μ*g/mL poly-L-lysine (PLL) (Sigma-Aldrich) in DMEM (Gibco by Invitrogen, Carlsbad, USA) and incubated at 37°C with 5% CO_2_ for 1 hour. The wells were washed three times with PBS. After washing, the prepared wells were filled with 170 *μ*L primary attachment medium, containing DMEM (Gibco), 10% fetal bovine serum (Sigma-Aldrich), 25 mM HEPES buffer (Gibco), and 300 U/mL penicillin (Sigma-Aldrich).

### 2.6. SGN Cell Culture

The spiral lamina containing the SG was carefully separated from the modiolus and immediately transferred into primary cell culture medium. It was then cut into equal 300 to 500 *μ*m portions and transferred to prepared culture plates. The first incubation period was 24 hours at 37°C in primary attachment medium. After that the culture medium was changed to serum-free maintenance media, containing DMEM (Gibco), 25 mM HEPES buffer (Gibco), 6 mg/mL glucose (Gibco), 300 U/mL penicillin (Sigma-Aldrich), and 30 *μ*g/mL N_2_ supplement (Gibco). The maintenance medium was supplemented with 10 ng/mL recombinant BDNF for trophic support of SG neuron survival and optimization of neurite outgrowth (R&D Systems, Minneapolis, MN, USA). Cultures were kept in a humidified incubator at 5% CO_2_ and 37°C for 72 h. In experimental cultures, 10 *μ*M or 100 *μ*M rapamycin (Sigma-Aldrich) was added to the maintenance media. Maintenance media without rapamycin (DMSO only) were used to treat control explants.

### 2.7. SGN Immunohistochemistry

Explants were fixed with 200 *μ*L 4% paraformaldehyde for 20 min at room temperature and washed twice with PBS (Gibco). After washing, the wells were filled with 200 *μ*L of permeabilization solution containing 5% triton X-100 (Sigma-Aldrich) for 10 min at room temperature. The wells were washed twice and then the first antibody (200 *μ*L of mouse monoclonal 200 kDa anti-neurofilament antibody diluted 1 : 500 in PBS) was added for labeling of the neurites. After overnight incubation at 4°C and an additional washing step, the wells were coated with 200 *μ*L of the secondary antibody solution against the species of the primary antibody (FITC-anti-mouse 1 : 100 diluted in PBS, Jackson Immunoresearch, West Grove, PA) for 2.5 hours at room temperature. Staining specificity was confirmed by a series of negative control staining without primary antibodies. 20 SG explants were studied for each condition.

### 2.8. Quantification of Neuronal Survival

To assess effects on neuronal survival, SG explants were cultured as described above for 72 h, except that the explants were grown on glass cover slips. The explants were fixed as above and treated with 0.5% peroxide in methanol to block endogenous peroxidases. Then explants were treated with a mouse monoclonal antibody IgG against rat neurofilament 200 (Sigma- Aldrich) followed by a biotinylated secondary anti-mouse IgG and developed by an avidin and DAB procedure (Vector Laboratories, Burlingame, CA, USA). The tissue was cleared with citrosol (Fisher Scientific, Waltham, MA, USA) to allow the visualization of the cell somas for evaluation of neuronal survival. 8 SG explants were studied for each condition.

### 2.9. SGN Data Analysis

Digital images for immunohistochemistry were obtained on a fluorescence microscope (Olympus IX71, Center Valley, PA, USA) and photographed with an AxioCam (Zeiss, San Diego, USA). Digital images for the quantification of neuronal survival were obtained on an inverted microscope (Olympus BX63, Center Valley, PA, USA). Images were optimized to achieve uniform brightness and contrast using Adobe Photoshop (Adobe Systems Inc., San Jose, CA, USA). Neurite outgrowth from the SG was evaluated by measuring the number and lengths of the processes. Growth of supporting cells was evaluated by measuring the area of the skirt surrounding the SGN. Images of the immunostained cultures were analyzed by using ImageJ software (NIH, Bethesda, MD, USA). Each neurite was traced, and number of neurites, average lengths of neurites, and area of the supporting cells per explant were analyzed. Neuronal survival was analyzed by evaluating the number of neurons per 100 *μ*m. Statistical analysis was performed using a one-way analysis of variance (ANOVA) followed by Tukey's least significant difference post hoc test with Bonferroni correction. Data presented in the text and figures are the means and standard deviations. Results were considered to be significant when the likelihood for a type 1 error was less than 5% (*P* < 0.05).

## 3. Results

### 3.1. Raptor, the Unique Adaptor Protein of mTORC1, Is Expressed in the Mammalian Cochlea

**Figure 1:**
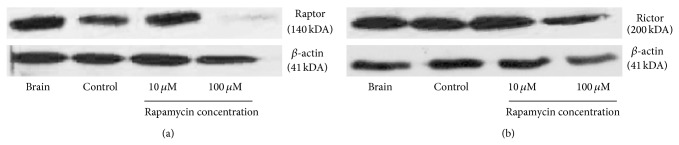
Representative western blots of raptor and rictor. Cochleae were either exposed for 24 hours to control media (control) or media containing rapamycin (10 *μ*M or 100 *μ*M, resp.). Brain tissue served as positive control. *β*-actin was used as an internal control.

Western blotting revealed that raptor, the unique adaptor protein of mTORC1, is expressed in the mammalian cochlea. Interestingly, cochleae exposed for 24 hours to the lowest concentration of rapamycin used in this study (10 *μ*M), control cochlea (culture media with DMSO only), and brain tissue (positive control) showed strong expression of raptor. However, cochleae exposed to the highest concentrations used in this study (100 *μ*M) showed only faint expression of raptor. *β*-actin was used as an internal control (Figure 1(a)).

### 3.2. Rictor, the Unique Adaptor Protein of mTORC2, Is Expressed in the Mammalian Cochlea

Western blotting revealed that rictor, the unique adaptor protein of mTORC2, is expressed in the mammalian cochlea. Cochleae exposed for 24 hours to the lowest and highest concentration of rapamycin used in this study (10 *μ*M and 100 *μ*M, resp.), control cochleae (culture media with DMSO only), and brain tissue (positive control) showed strong expression of rictor. *β*-actin was used as an internal control (Figure 1(b)).

### 3.3. Inhibition of mTOR by Rapamycin Results in a Dose Dependent Toxic Effect on HCs

**Figure 2:**
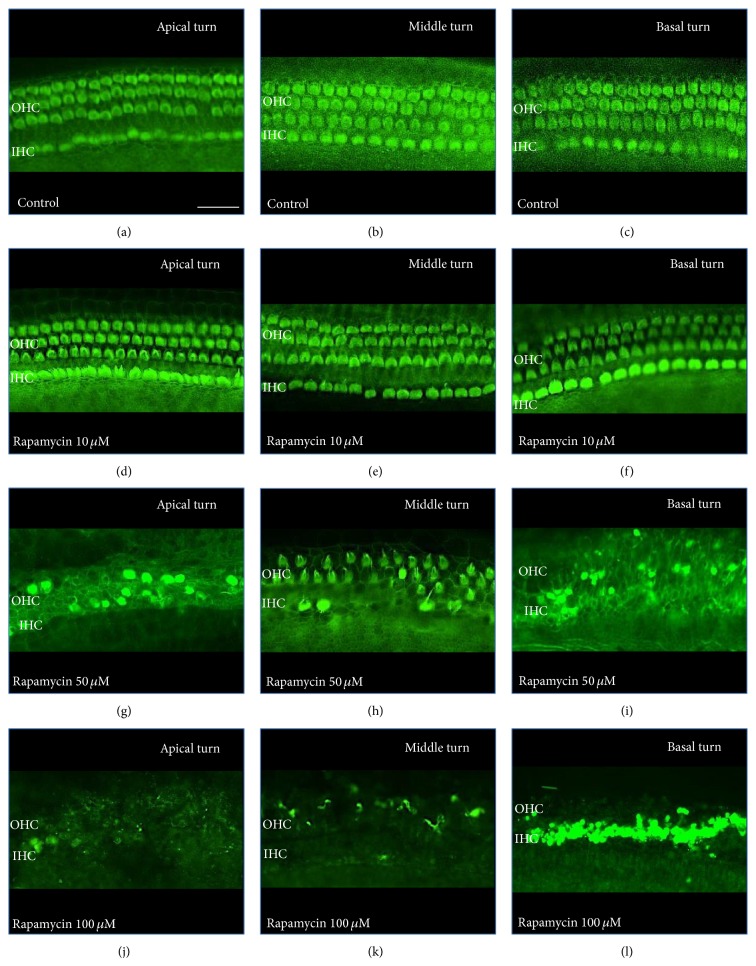
Effect of rapamycin on auditory HCs by cochlear turns. Photograph of anti-myosin VIIa-labeled OCs. Scale bar 20 *μ*m. (a–f) Untreated OCs (control) and OCs exposed to 10 *μ*M rapamycin demonstrate three rows of OHCs and a single row of IHCs in all cochlear turns. (g–l) OCs treated with increasing concentrations of rapamycin (50 *μ*M and 100 *μ*M, resp.) show a dose dependent loss of OHCs and IHCs.

**Figure 3:**
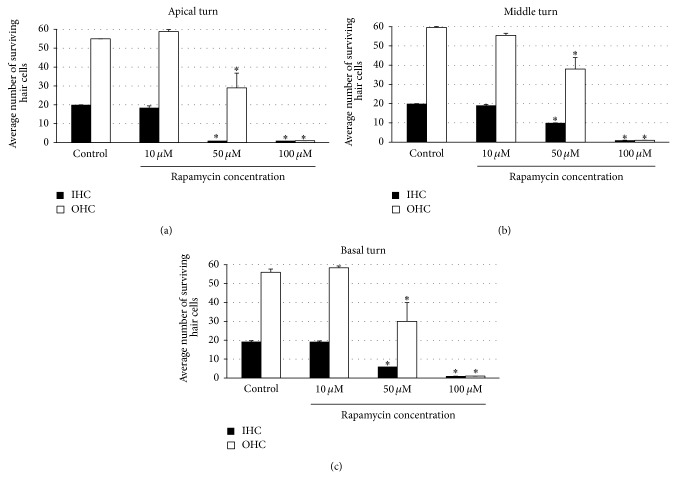
Quantitative analysis of surviving OHCs and IHCs treated with rapamycin by cochlear turns. (a) Average surviving OHCs and IHCs in the apical turn with increasing concentrations of rapamycin compared to control. (b) Average surviving OHCs and IHCs in the middle turn with increasing concentrations of rapamycin compared to control. (c) Average surviving OHCs and IHCs in the basal turn with increasing concentrations of rapamycin compared to control. There was a dose dependent statistically significant decrease in IHC and OHC survival in OCs treated with increasing concentrations of rapamycin in all cochlear turns. Asterisks indicate significant difference compared to control (*P* < 0.05). Data are expressed as the mean number of surviving OHCs and IHCs corresponding to 20 IHCs. Vertical lines represent one standard deviation. *n* = 6 for each experimental condition.

Treatment with rapamycin showed a dose dependent HC loss in all cochlear turns (Figures 2 and 3). Control explants and explants treated with the lowest dose used in this study (rapamycin 10 *μ*M) showed three orderly rows of outer hair cells (OHCs) and a single row of inner hair cells (IHCs) in all cochlear turns. We observed a statistically significant loss of IHCs and OHCs with increasing concentrations of rapamycin (50 *μ*M and 100 *μ*M, resp.) in all cochlear turns (ANOVA, *P* < 0.05 for all conditions). A total loss of hair cells (IHCs and OHCs) was found in explants treated with the highest concentration of rapamycin used in this study (100 *μ*M) in all cochlear turns.

### 3.4. Inhibition of mTOR by Rapamycin Significantly Reduces Number of Neurites per SGN

**Figure 4:**
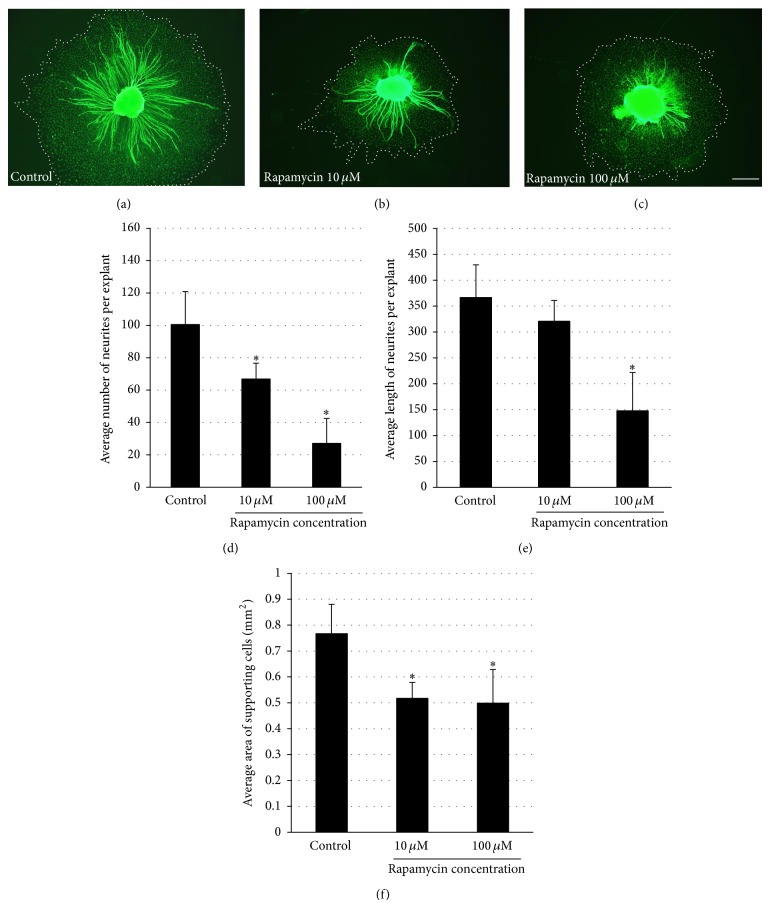
Effect of rapamycin on SG explants and their supporting cells. (a–c) Representative SG explants stained with anti-200 kDa neurofilament antibody after treatment with DMSO (control), 10 *μ*M rapamycin, and 100 *μ*M rapamycin. Scale bar 300 *μ*m. (d) Average number of SG neurites observed per SG explant. The numbers of neurites observed in control samples are compared to those seen with two different concentrations of rapamycin. Rapamycin-treated samples showed significantly less SG neurites per SG explant compared to control in both concentrations used. (e) Average length of SG neurites observed on SG explants. The lengths of neurites observed in control samples are compared to those seen with two different concentrations of rapamycin. Rapamycin-treated samples were significantly shorter in the highest rapamycin concentrations used. (f) Average area of supporting cells observed around SG explants. Rapamycin treatment resulted in significantly smaller area of supporting cells around the SG explant compared to control samples in both concentrations used. Asterisks denote statistical difference compared to control (*P* < 0.05). Data are represented as mean. Vertical lines represent one standard deviation. *n* = 20 for each experimental condition.

The influence of inhibition of mTOR by rapamycin on SG neurite formation is illustrated in Figure 4. Rapamycin treatment results in decreased number of neurites per SG explant in both concentrations used in this study (10 *μ*M and 100 *μ*M rapamycin, resp.) compared to control explants (ANOVA, *P* < 0.05 for both conditions).

### 3.5. Inhibition of mTOR by Rapamycin Significantly Reduces SG Neurite Length

Rapamycin treatment reduced SG neurite length in a dose dependent manner (Figure 4). SGN treated with the highest concentration of rapamycin used in this study (100 *μ*M) showed a statistically significant decrease of SG neurite length compared to control (ANOVA, *P* < 0.05).

### 3.6. Inhibition of mTOR by Rapamycin Results in Decreased Area of Supporting Cells

Rapamycin also significantly decreased the area of nonneuronal cells, which have been previously identified as fibroblasts and Schwann cells [24], growing around the explant, as compared to the negative control (Figure 4, ANOVA, *P* < 0.05).

### 3.7. Inhibition of mTOR by Rapamycin Does Not Influence Neuronal Survival

**Figure 5:**
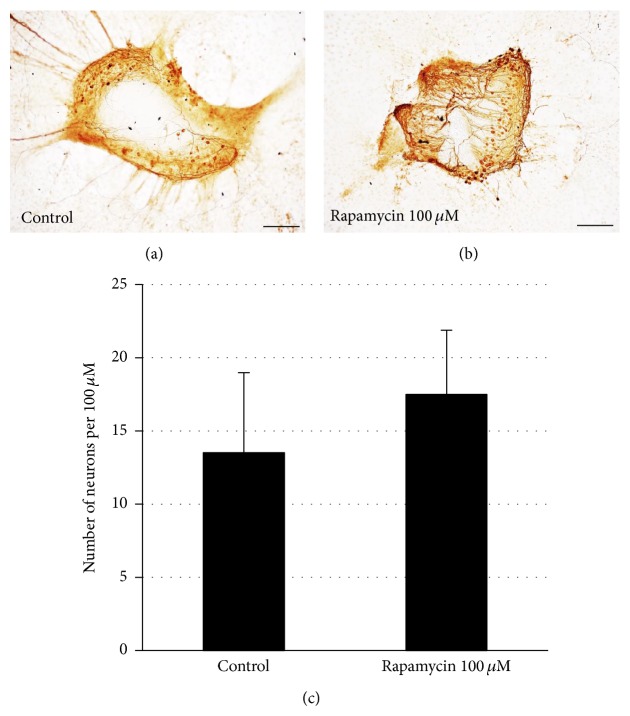
Effects of rapamycin on SG neuronal survival. (a-b) Representative SG explants after treatment with DMSO only (control) and rapamycin 100 *μ*M. (c) Rapamycin treatment did not show a difference in SGN survival compared to controls (*P* > 0.05). Scale bar 100 *μ*m. Data are represented as mean. Vertical lines represent one standard deviation. *n* = 8 for each experimental condition.

The decreased number of neurites extending from SG explants could reflect the altered survival and/or neuritogenesis of SGNs. To assess this, we evaluated the survival of SGN cell bodies within explants treated with the highest dosage of rapamycin used in this study (100 *μ*M). Rapamycin did not influence SG neuronal survival when compared to controls. (Figure 5, ANOVA, *P* > 0.05).

## 4. Discussion

At present, limited information about the role of mTOR in the mammalian inner ear is available. The aim of our study was to investigate the effects of the mTOR inhibitor, rapamycin, in the mammalian inner ear* in vitro*. We found that rapamycin treatment results in dose dependant HC damage* in vitro*. Moreover, SGN treated with this drug showed a significant reduction in SGN neuritogenesis and dendrite formation* in vitro.*

First, we demonstrated by performing western blotting that the two independent mTOR complexes are present in the mammalian cochlea. This was done by using antibodies against the unique adaptor proteins, raptor for mTORC1 and rictor for mTORC2. Cochleae exposed to the lowest concentration of rapamycin used in this study (10 *μ*M) and control cochleae (culture media with DMSO only) showed strong expression of raptor. However, cochleae exposed to the highest concentrations used in this study (100 *μ*M) showed only faint expression of raptor. In contrast, we observed no difference in the expression of rictor in cochleae treated with increasing concentrations of rapamycin compared to control. This is in line with the observation that rapamycin primarily inhibits mTORC1 and only prolonged exposure to rapamycin influences mTORC2 function [25].

Our study showed a dose dependent toxic effect of rapamycin on HCs* in vitro*. In contrast to our results, Fang and Xiao [22] showed an otoprotective effect of rapamycin in cisplatin-induced HC damage in adult rats* in vivo*. In the study by Fang and Xiao, rapamycin treatment improved tone burst auditory brainstem response (ABR) and reduced the level of oxidative products in cisplatin treated animals, whereas animals treated with rapamycin alone did not show significant differences in the ABR measurements compared to controls. However, how can we explain the fact that rapamycin protects from cisplatin-induced HC toxicity* in vivo*, while it damages HCs and SGNs* in vitro*? There were significant differences between the study conducted by Fang and Xiao and our* in vitro* model. We used in our* in vitro *experiments cochlear explants harvested from 5-day-old animals. Fang and Xiao used 8-week-old adult Wistar rats. It is known that younger animals are more sensitive to ototoxins [1, 3]. Fang and Xiao injected 2 mg/kg rapamycin intraperitoneally. However, the concentration of rapamycin that reached the plasma or the inner ear is unknown. One explanation might be that the concentration of rapamycin that reached the inner ear in the study by Fang and Xiao was below the dosage for toxicity. However, Fang and Xiao show an otoprotective effect on cisplatin-induced HC damage. The authors explained the effect observed by decreased oxidative stress and promoted autophagy. In contrast, our results suggest increased oxidative stress after rapamycin treatment resulting in HC damage* in vitro*. Interestingly, interactions between rapamycin and cisplatin are known. It is described that rapamycin can influence the effect of cisplatin and sensitize cells through downregulation of antiapoptotic proteins like p53. In the literature, it is described that mTOR inhibitors enhance the anticancer therapy with cisplatin [26, 27]. These observations and our results caution against the use of rapamycin cotreatment with cisplatin and confirm the need for additional studies in order to evaluate these conflicting results.

How can the toxic effect of rapamycin in our study be explained? Rapamycin inhibits mTORC1 and blocks the phosphorylation of its downstream targets. This results in cell cycle progression and apoptosis [28]. The treatment with low-dose concentrations of rapamycin (1 ng/mL, 5 ng/mL, and 10 ng/mL) demonstrated in two human HCC (hepatocellular carcinoma) cell lines a significant increase in the rate of apoptosis and decrease in their synthesis rate [29]. High-dose rapamycin (20 *μ*M) results in complete G1 arrest in human cancer cells [30]. Our experiment demonstrates a dose dependent damage of the auditory HCs. Low-dose concentrations of rapamycin (10 *μ*M) had no toxic effect, while 50 *μ*M and 100 *μ*M rapamycin result in dose dependent HC loss. Montcouquiol and Corwin [31] showed that incubation of vestibular epithelia from 2-day-old rats with rapamycin resulted in a dose dependent inhibition of S-phase entry. In line with our results, Cai et al. [32] demonstrated an inhibition of cell proliferation and cell cycle and promoted apoptosis in retinal pigment epithelial cells after rapamycin treatment.

Our study indicates that mTOR inhibition by rapamycin treatment not only negatively affects auditory HCs but also influences SGNs. Rapamycin treatment significantly decreases number of neurites per SGN and reduces SGN neurite length. Interestingly, we observed no difference in neuronal survival indicating that the reduction in number of neurites per SGN reflects decreased neurite formation. Our results suggest that mTOR might be involved in neurite formation and neurite outgrowth in the cochlea. Two different subtypes with different functions and cellular interactions of SGNs are known, type I and type II SGNs [33]. The dendrites of type I cells built afferent synapses exclusively with the IHCs, while the dendrites of type II cells exclusively interact with the OHCs [33]. It should be noted that in the present study we could not distinguish between type I and type II SGNs. 95% of SGNs are type I cells; therefore it seems likely that this subtype of neuron dominates our results.

In our experiments, we used organotypic explants from cochlear SGNs. Besides neurons, the explants contained supporting cells, including fibroblasts and Schwann cells, which reside between the neurons* in vivo*. Both cells are known to provide guidance cues to advancing neurites and growth cones and, therefore, might have influenced the observed reduction in neuritogenesis and length of neurites in our experiments. SG explants treated with rapamycin showed not only decrease in number of neurites per SG explant and reduction in length of neurites but also a significant decrease of the area of the supporting cells surrounding the SG explant. The inhibition of nonneuronal cell growth was somewhat less than the inhibition of neurite length. However, this finding suggests that the reduction of SG neurite elongation due to rapamycin may be mediated, at least in part, via Schwann cells or fibroblasts. However, the possibility of directly mediated effects must still be considered.

## 5. Conclusion

In summary, our data indicate that mTOR inhibition by rapamycin treatment results in HC loss and decreases SGN neuritogenesis and dendrite length of SGNs* in vitro*. These findings suggest that mTOR might be involved in HC survival, SGN neuritogenesis, and dendrite extension in the mammalian cochlea.
